# Plasticity of Adult Sensorimotor System

**DOI:** 10.1155/2012/768259

**Published:** 2012-12-24

**Authors:** Marie-Hélène Canu, Jacques-Olivier Coq, Mary F. Barbe, Hubert R. Dinse

**Affiliations:** ^1^Université Lille Nord de France, 59000 Lille, France; ^2^Laboratoire “Activité Physique, Muscle et Santé,” EA 4488, IFR 114, Université Lille 1, Sciences et Technologies, 59650 Villeneuve d'Ascq, France; ^3^Faculté de Médecine-Secteur Nord, UMR1072 UNIS Inserm/Aix-Marseille Université, Bat C, Bd Pierre Dramard, 13344 Marseille Cedex 15, France; ^4^Comprehensive NeuroAIDS Center, Animal Models and Behavioral Testing Core, Temple University School of Medicine, 3500 North Broad Street, Philadelphia, PA 19140, USA; ^5^Neural Plasticity Lab, Institut fur Neuroinformatik, Ruhr University Bochum, Building NB 3, 44780 Bochum, Germany

The central nervous system is a highly plastic structure that can adapt throughout the lifespan of higher mammals to changes in environment. The establishment of neural connectivity, during embryonic period and during early postnatal stages, is under genetic and epigenetic control. Environmental factors, in particular during the critical period, can heavily influence the organization of the brain, both in the short and long term. In the 1980s, Kaas, Merzenich, and collaborators have reported that the adult central nervous system of mammals was also capable of a remarkable reorganization following traumatic lesions (amputation, nerve section, etc.). Since these pioneering works, the concept of neural plasticity stirs up a growing interest in the scientific community. Many works have been focused on the extensive reorganization of cortical and subcortical structures in relation to individual experience (*activity-dependent plasticity*). Within the sensorimotor system, plastic mechanisms can occur spontaneously after an injury, such as stroke or spinal cord injury (*injury-induced plasticity*). These changes can sometimes be considered as adaptative or compensatory, but in some cases, they may lead to a maladaptative process. More recently, it has been demonstrated that in addition to training, practicing, and use, mere exposure to repetitive sensory or central stimulation, significant changes of behavior and neural processing can be evoked. In patients, one can take advantage of both the activity-dependent plastic properties of the CNS to develop efficient therapeutic methods, in particular through repetitive sensory or direct current/magnetic stimulation. Increasing the basic knowledge of activity-dependent plasticity throughout the lifespan of humans can substantially influence the treatment and rehabilitation methods used for a variety of movement disorders.

Four contributions to this special issue provide an overview of data in the field of activity-dependent plasticity of adult sensorimotor system. They are accompanied by a research paper addressing the question of adaptation to prisms. All papers pay a particular attention to rehabilitation strategies.

The research paper is entitled “*Left-deviating prism adaptation in left neglect patient: reflexions on a negative result*” by J. Luauté et al. In healthy subjects, optical prisms are used to induce experimentally a sensorimotor plasticity. Prism adaptation is also used in patients with left spatial neglect, where it appears as a promising therapeutic intervention for rehabilitation. The authors demonstrate that there is a directional specificity of the prisms: patients with left spatial neglect are not affected by prism adaptation to a leftward optical shift whereas right-deviating prisms are known to have beneficial effects. The paper also raises the question about the conditions necessary to produce cognitive after-effects. Very interestingly, it shows that no cognitive effects are found in the absence of adaptation, playing against the hypothesis that active exposure to a simple modification of sensorimotor coordinates by simple visuomanual pointing is sufficient to reduce left spatial neglect.

This paper is followed by four reviews. The contribution by A. Sterr and A. B. Conforto “*Plasticity of adult sensorimotor system in severe brain infarcts: challenges and opportunities*” is a very integrative overview of the sensorimotor system. Interestingly, it highlights that the execution of movement should be considered from anticipatory processes within cortical areas to motor performance and that treatment strategies should consider all these aspects. In addition, it points out that use-dependent reorganization of neural representations is a complex process, which is modulated by various factors, such as attention, motivation, tiredness and fatigue, and engagement of the patient with the therapy process.

The other reviews are focused on several levels of the motor control: cortical, spinal, or corticospinal drive. Although reflexes had been thought to be very stereotyped for a long time, it is now well established that spinal reflex characteristics can change in response to an operant conditioning task, providing evidence for activity-induced plastic changes within the spinal cord. In consequence, the stretch reflex or the H-reflex has been extensively studied to evaluate spinal plasticity in the mature nervous system. The paper by B. Tahayori and D. M. Koceja “*Activity-dependent plasticity of spinal circuits in the developing and mature spinal cord*” compares plasticity of spinal circuits during development and in adulthood. In childhood, the authors point out the role of input from both large-diameter primary afferents and from supraspinal afferents. The paper also explains how changes in the reflex pathway occur and how they become permanent and highlights the role of presynaptic inhibition of afferent fibers.

In her review “*Plasticity of corticospinal neural control after locomotor training in human spinal cord injury*” M. Knikou provides an overview of cortical control of spinal cord after training. The author demonstrates that the spinal cord circuitries have the capacity to alter their structure and function with motor training, in particular in spinal cord-injured patients. Rehabilitation protocols should take advantage of the plastic capabilities of corticospinal drive and spinal interneuronal circuits for restoration of locomotion after an injury.

An effective motor rehabilitation in humans requires a better knowledge of somatosensory physiology and its influence on primary motor cortex (M1) activity. Primary somatosensory cortex (S1) and M1 have anatomical and functional connections. In humans as in animals, there is substantial evidence that direct manipulation of S1 activity alters motor behavior. M. Jacobs et al. “*Plasticity-inducing TMS protocols to investigate somatosensory control of hand function*” provide a critical review showing that plasticity-inducing TMS protocols are a powerful tool to modulate S1 physiology, tactile perception, and neural activity within both S1 and M1 and suggest that these protocols might be used to improve hand function in patients.

We hope that this special issue will have brought new insights into the underlying mechanisms of neural plasticity that occurs in response to changes in sensorimotor experience and will help to elaborate efficient rehabilitation strategies and/or to optimize the currently available strategies.

*Marie-Hélène Canu* *Marie-Hélène Canu* *Jacques-Olivier Coq* *Jacques-Olivier Coq* *Mary F. Barbe* *Mary F. Barbe* *Hubert R. Dinse* *Hubert R. Dinse*

